# Pathophysiological mechanisms of gonadotropins– and steroid hormones–related genes in etiology of polycystic ovary syndrome

**DOI:** 10.22038/ijbms.2018.31776.7646

**Published:** 2019-01

**Authors:** Zahra Shaaban, Arezoo Khoradmehr, Mohammad Reza Jafarzadeh Shirazi, Amin Tamadon

**Affiliations:** 1 Department of Animal Science, College of Agriculture, Shiraz University, Shiraz, Iran; 2 Research and Clinical Center for Infertility, Yazd Reproduction Sciences Institute, Shahid Sadoughi University of Medical Sciences, Yazd, Iran; 3 The Persian Gulf Marine Biotechnology Research Center, The Persian Gulf Biomedical Sciences Research Institute, Bushehr University of Medical Sciences, Bushehr, Iran

**Keywords:** Genes, Gonadotropins, Hormones, Hyperandrogenism, Polycystic ovary syndrome, Physiopathology, Steroids

## Abstract

**Objective(s):**

Polycystic ovary syndrome (PCOS) is an endocrinopathy in women, which, unlike its impact on fertility and health of women, there is no clear understanding about the causal mechanisms of this pathogenesis. The aim of this review paper is to investigate the pathophysiological pathways affecting the PCOS etiology, based on functions of gonadotropins– and steroid hormones–related genes.

**Materials and Methods:**

Due to different hormonal and metabolic signs of this complex disorder, different hypotheses are mentioned about etiology of this syndrome. Because of the heterogeneity of the reasons given for this syndrome and the spread of the effective genes in its pathophysiology, most of genes affected by sex-related hormonal imbalances are examined for discriminative diagnosis. For this purpose, published articles and reviews dealing with genetic evaluation of PCOS in women in peer-reviewed journals in PubMed and Google Scholar databases were included in this review.

**Results:**

In previous studies, it has been well demonstrated that PCOS in some individuals have a genetic origin. Pathophysiological functions of genes are primarily responsible for the synthesis of proteins that have role in PCOS before hyperandrogenism including *GnRHR, FSHβ, FSHR, LHCGR, CYP19A1, HSD17B, AR* and *SHBG*, and their effects in PCOS of human have been confirmed.

**Conclusion:**

Hormonal imbalances are the first reason mentioned in PCOS etiology, and usually characterized with menstrual irregularities in PCOS women. Hyperandrogenism and gonadotropin secretion disorders are shown in PCOS condition, which are related to steroidogenesis pathways and hypothalamic–pituitary–ovarian axis disturbances, respectively.

## Introduction

 Polycystic ovary syndrome (PCOS) is a multifactorial disorder and different genetic, hormonal, and environmental etiology can contribute to its pathology (1). The diagnosis of PCOS in women is usually according to the ESRHE/ASRM criteria known as Rotterdam criteria, which is based on having at least two of three characteristics of oligo-ovulation/anovulation, hyperandrogenism and polycyclic ovaries using ultrasonographic images (2). According to Androgen Excess Society (AES) criteria, clinical or biochemical diagnosis of hyperandrogenism is required simultaneously with an oligo-ovulation/anovulation, or ultrasound images of polycyclic ovaries (3).

Proof of genetic origin of hyperandrogenism in PCOS is performed by familial aggregation studies as well as identification of dependent genetic variants that are associated with PCOS susceptibility (4). Prevalence of PCOS among various ethnic and racial groups was similar, unlike the apparent differences in phenotypic attributes among different populations that affect five to seven percent of women in reproductive age (5). Therefore, it is thought that similar genes or genetic networks can affect the incidence of PCOS among various populations (6). 

Many hypotheses about the pathophysiology of PCOS that have been explained so far, include: resistant to rupture of follicles due to shell thickness, ovarian hyperandrogenism, luteinizing hormone (LH) hypersecretion, hyperinsulinemia, and impaired ovarian follicular development due to increased follicular development blocker paracrine factors, such as anti-Müllerian hormone (AMH) (4). The causes of any of these abnormalities may be due to genetic factors that are commonly found in homozygous twins studies or due to metabolic, hormonal, nutritional or even toxic changes during embryonic development and in early stages of female gonad differentiation (4). But still, the exact origin of each abnormality is unclear. Several observations show that the interaction of several genetic factors and environmental factors are necessary for PCOS development (7).

In this review, we attempted to explain the pathophysiological function of PCOS candidate genes, effective in sex-related hormonal disorders, which have been studied so far, and then summarize the pathophysiological pathways that are influenced by gonadotropins– and steroid hormones–related genes. So, there may be a new insight into identifying the causes of PCOS development. 

For this purpose, published articles and reviews dealing with genetic evaluation of PCOS in women in peer-reviewed journals in PubMed and Google Scholar databases were included in this review. The searches were performed by using keywords mentioned in the MeSH regarding genetic studies of PCOS. In combination with PCOS, gene and women, these keywords were used to specify the searching results: human, patient, pathogenesis, hyperandrogenism, and gonadotropin.

**Physiological hypotheses on the prognosis of PCOS**

**Figure 1:**
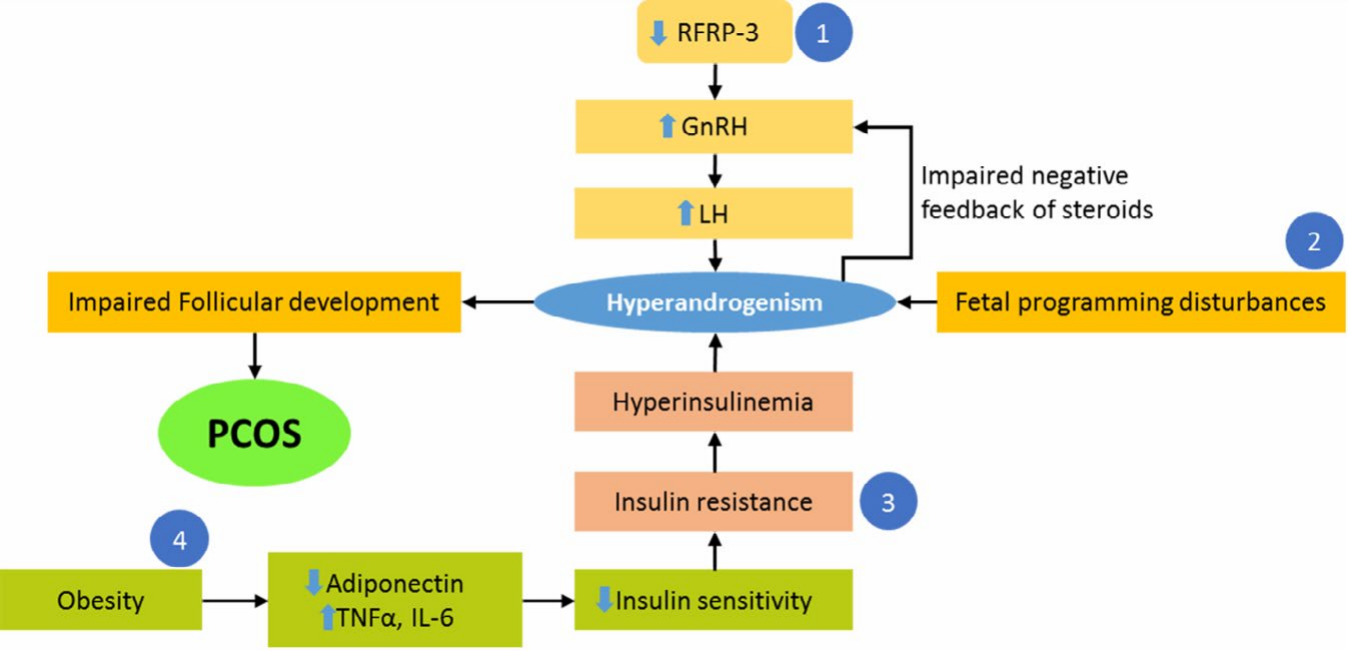
The pathophysiological pathways that presumed to mediate polycystic ovary syndrome (PCOS) formation are expressed. Four different pathophysiological pathways lead to PCOS. Abbreviation: RFRP-3, arginine-phenylalanine-amide (RFamide) -related peptide 3; GnRH, gonadotropin releasing hormone; LH, luteinizing hormone; TNFα, tumor necrosis factor-α; IL-6, Interleukin-6

Despite many investigations on PCOS and the expression of different hypotheses about the development of PCOS, the main cause of this syndrome is still unknown. The PCOS is a syndrome with different and completely heterogeneous characteristics; therefore, there are different pathways that may be involved in its etiology. For instant, a) hormonal imbalances such as hyperandrogenism increased LH/ follicle stimulating hormone (FSH) ratio, increased estrogen levels, and decreased serum progesterone, b) reproductive disorders such as non-ovulation, and menstrual irregularities, c) metabolic abnormalities such as impaired glucose tolerance and insulin resistance, obesity, cardiovascular disease, and type 2 diabetes, and d) changes in serum lipid parameters, are all components of this complex syndrome. Naturally, the appearance of each of these phenotypic traits follows a special physiological pathway in the body (Figure 1), but which pathway(s) causes the disease and which pathway is affected after the disease, is still in debate. Below, two hypotheses are explained based on the two groups of genes.

***Hypothalamus-pituitary-ovarian axis***

The elevation of frequency and amplitude of the release of gonadotropin releasing hormone (GnRH) and subsequent LH secretion is the most important pathophysiological feature of PCOS (8). It seems that the most important reason for GnRH secretion impairment is dysfunction of gonadotropin-inhibitory hormone (GnIH). In our previous study, induction of PCOS by continuous light reduced the mRNA expression of arginine-phenylalanine related peptide-3 (RFRP-3) neuropeptide in rats (9). So, in PCOS women, this disorder may occur and can be examined through regular daily or hourly evaluations of serum samples, and examination of LH pulse secretion, which is clearly indicative of GnRH release and the reduction of inhibitory effect of RFRP-3 neurons. Therefore, by using RFRP-3 agonist drugs in PCOS patients, LH secretion can be reduced and PCOS symptoms may ameliorate; meanwhile, this mode indicates that the activity of RFRP-3 neurons dropped. 

***Hyperandrogenism***

**Figure 2:**
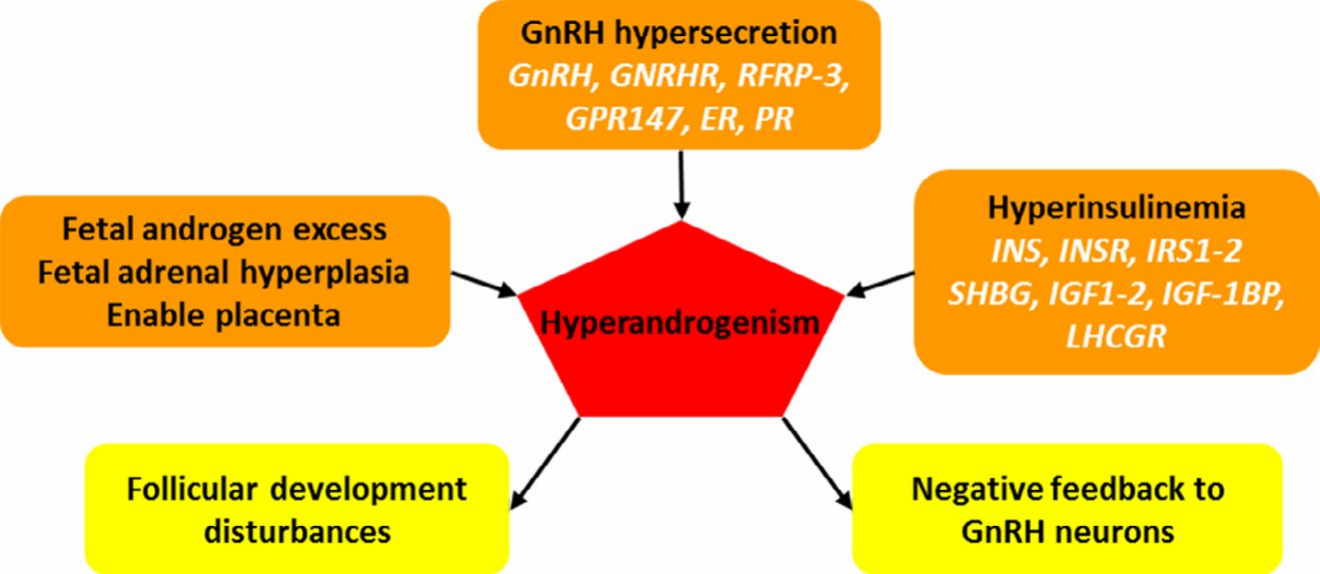
Relationship between the pathogenesis of polycystic ovary syndrome (PCOS) and steroid hormones and the effective genes in functioning of steroid hormones. Hyperandrogenism causes its adverse effects on follicular development and negative feedback mechanisms. Abbreviation: ER, estrogen receptor; GnRH, gonadotropin releasing hormone; GnRHR, GnRH receptor; GPR147, G protein-coupled receptor 147; IGF, insulin-like growth factor; IN, insulin; INSR, insulin receptor; IRS, insulin receptor substrate; LHCGR, luteinizing hormone/chorionic gonadotropin receptor; PR, progesterone receptor; RFRP-3, arginine-phenylalanine-amide (RFamide)-related peptide 3; SHBG, sex hormone-binding globulin

Another important endocrine feature of PCOS is the increased level of serum androgens. This hypothesis that PCOS can be due to androgen hypersecretion and eventually hyperandrogenism, for the first time was expressed in 1989-1955 (10). Hyperandrogenism can occur for several reasons, and it can disrupt normal activity of ovary and interfere with menstrual cycle (Figure 2). The first reason, based on the above hypothesis, is the disruption of hypothalamic-pituitary axis and increase of LH secretion. LH affects ovarian theca cells and increases synthesis of androgens (8). The second reason is the metabolic abnormalities in PCOS such as insulin resistance and hyperinsulinemia (11). Hyperinsulinemia increases the secretion of androgens with different effects on ovary, adrenal, pituitary, LH receptor, sex hormone-binding globulin (SHBG) protein, etc. Another reason for hyperandrogenism is the exposure to androgens during fetal development, which can result in PCOS phenotypes in adulthood (4). During development of fetus, embryo may receive additional androgens for four reasons, resulting in epigenetic changes leading to PCOS in the future. Firstly, the mother has PCOS and placenta is also unable to perform aromatization and increase the concentration of SHBG, which will result in receiving maternal androgen via the placenta by fetus (12, 13). Second, the fetus has a genetic disorder, and the fetal undifferentiated ovary is the source of excess androgen production (4). The third reason is malformation of tissues producing androgens including the adrenal; for example, adrenal hyperplasia can also affect production of additional androgen. The fourth reason is hypothalamic-pituitary axis disorders during embryonic development simultaneously with evolution of this system that may increase androgen production (4). Therefore, the increase in serum androgen levels in both embryonic and adulthood plays an important role in initiating PCOS. However, the evaluation of two previous pathways, the hypothalamus-pituitary axis and insulin pathways, are valuable.

PCOS phenotypes and its related genes

PCOS is a complex syndrome and heterogeneous disturbance with a prominent influence of both genetic and environmental factors (14). Familial clustering studies of PCOS suggest the strong effect of genetic factors on PCOS pathogenesis (15). Hereditary background of PCOS is well-documented based on family studies and phenotypic attributes (16). PCOS is an oligogenic trait and has about 70% heritability (17). The PCOS genetic origin evidence, based on previous research findings, include familial aggregation, male phenotype, and twin studies (7). Although, there is an increased incidence of PCOS phenotypic traits such as hyperandrogenism, and type 2 diabetes mellitus among first-degree relatives, but the mode of heredity is unknown (14). 

The genetic evaluation of PCOS was mainly conducted through means that have the candidate gene approach with focusing on gene selection based on its assumed role in the syndrome; but, this approach has failed in complex syndrome such as PCOS, due to incomplete understanding of PCOS pathophysiology, and access to one or more markers of interest gene (18). Hence, in new methods for recognition of causal genes, the use of methods like genome wide association study (GWAS), as well as large case-control groups from several thousands of participants, has been able to found several variants associated with PCOS (4). At the first time, in GWAS study of PCOS in Han Chinese women for discovering the loci associated with PCOS, between the PCOS and control women, the association between PCOS and three locus 2p16.3 (rs13405728), 2p21 (rs13429458) and 9q33.3 (rs2479106) has been firmly identified (19). In another group of Han Chinese women, in addition to the three locus related to PCOS, seven new PCOS genetic variants at 9q22.32, 11q22.1, 12q13.2, 12q14.3, 16q12.1, 19p13.3, and 20q13.2 and two non-dependent signals in 2p16.3 (*FSHR* gene) were identified (20). These findings were later confirmed in studies in Europe (21, 22), United State (18, 23), and other parts of the world with different racial diversity (4).

These genes are primarily responsible for the synthesis of proteins that belong to six categories, separated by pathophysiological function: a, gonadotropin secretion and actions, b, steroid hormones biosynthesis and functions, c, insulin secretion and signaling, d, insulin resistance and type 2 diabetes mellitus, e, obesity and dyslipidemia, f, chronic inflammatory reactions. In this article, the genes related to a, and b categories are examined.

**Figure 3:**
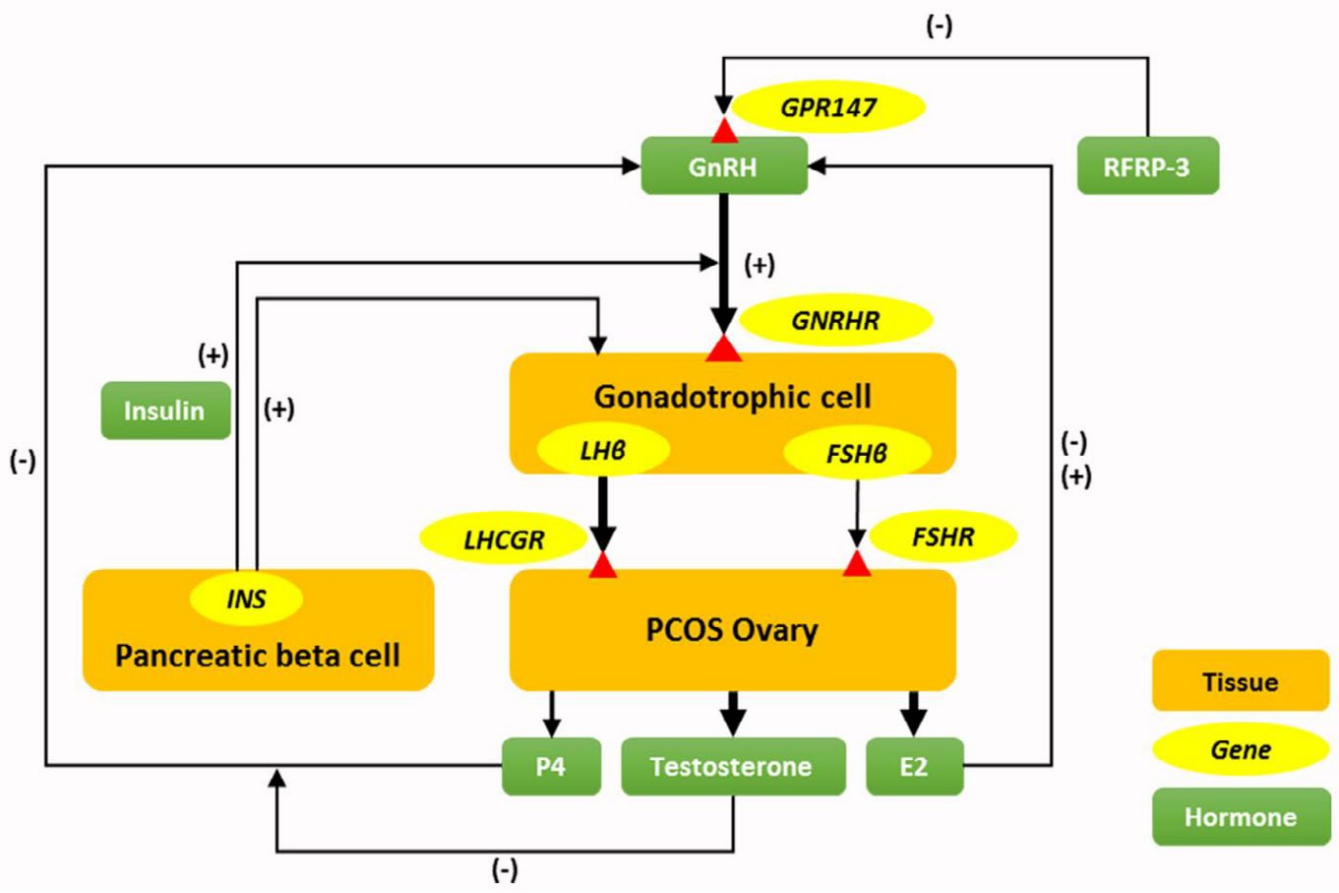
Relationship of polycystic ovary syndrome (PCOS) pathogenesis and four hypotheses have been suggested that explain impact of gonadotropin hormones on brain function in pathogenesis of PCOS. Elevation of gonadotropin releasing hormone (GnRH) pulses in PCOS condition may be due to reduction of arginine-phenylalanine related peptide-3 (RFRP-3) neuronal activity, incremental effect of hyperinsulinemia on GnRH release, or pituitary responsiveness to GnRH, and impaired the negative feedback of progesterone (P4) on GnRH neurons by androgen excess. Abbreviations: E2, Estradiol 2; FSH, follicle stimulating hormone; FSHR, follicle stimulating hormone receptor; INS, insulin gene; GnRHR, Gonadotropin releasing hormone receptor; GPR147, G-protein coupled receptors; LH, luteinizing hormone; LHCGR, luteinizing hormone/chorionic gonadotropin receptor; (-), Negative feedback; (+), Positive feedback

**Figure 4.**
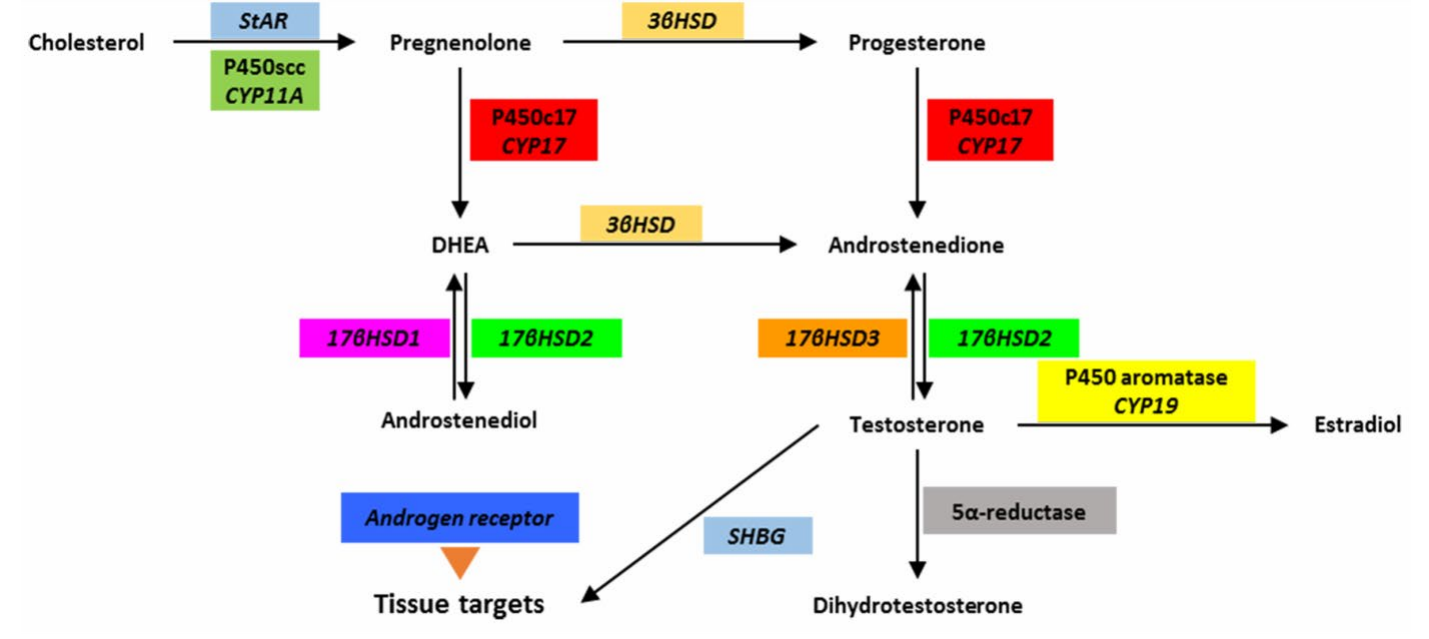
The steroidogenesis pathway and effective catalytic enzymes along with their coding genes. Abbreviations: CYP, cytochrome P450; DHEA, dehydroepiandrosterone; P450scc, cytochrome p450 side chain cleavage; SHBG, sex hormone-binding globulin; StAR, transporter protein that transmits cholesterol; βHSD, β-hydroxysteroid dehydrogenase

**Table1:** Candidate genes involve in etiology of polycystic ovary syndrome (PCOS) related to gonadotropin secretion and actions

**Gene** *	**Genetic marker(s) **	**Type of study**	**Physiologic function**	**Studied population**	**Type of polymorphism**	**Reference**
***GnRHR*** †	rs104893836	GWAS	Regulation of gonadotropin secretion	Israeli	ND	(35)
***GnRHR***	3′-UTR variant	GWAS	Regulation of gonadotropin secretion Stimulation of TSH secretion Insulin activity	Chinese Han	rs1038426	(37)
***LHβ***	Trp28Arf/Ile35Thr LH*β* variant	Case-control	Steroidogenesis regulation	Brazilian	rs1800447 rs34349826 Trp28Arg Ile35Th	(127)
***LHβ***	G1502A	Case-control	Steroidogenesis regulation	Egyptian	G1052A	(128)
***FSHβ***	chr 8p32.1 chr11p14.1 chr 9q22.32	GWAS	Folliculogenesis	European	chr 11p14.1 SNP rs11031006	(39)
***FSHβ***	Susceptibility loci 8p32.1 11p14.1 9q22.32 rs11031010	GWAS	Folliculogenesis	Chinese Han	rs11031010	(33)
***FSHR*** ***FSHβ*** ***LHCGR*** ***LHβ*** ***ESR1*** ***ESR2***	Genotype and allele frequencies and SNPs	Case-control	Steroidogenesis regulation Folliculogenesis Ovulation and female phenotypes	Pakistani	LHCGR FSHR ESR1	(129)
***LHCGR***	2p16.3	Case-control	Steroidogenesis regulation	European	No association	(48)
***LHCGR***	rs13405728	Case-control	Steroidogenesis regulation	Caucasian	No association	(49)
***LHCGR***	G935A, and ins18LQ	Case-control	Steroidogenesis regulation	Egyptian	G935A	(128)
***LHCGR***	G935A	Case-control	Steroidogenesis regulation	Egyptian	G935A	(130)
***LHCGR***	rs2293275	Case-control	Steroidogenesis regulation	Indian	rs2293275	(131)
***FSHR***	rs12994034	Case-control	Folliculogenesis	European	Yes	(23)
***FSHR***	Thr307Ala Asn680Ser	Meta-analysis	Folliculogenesis	Different	No association	(44)
***FSHR***	rs1394205 rs6165 rs6166	Case-control	Folliculogenesis	South Indian	rs6166	(45)
***FSHR***	rs2268361-T	Case-control	Folliculogenesis	European Greek	rs2268361-T in an intron of FSHR	(46)
***FSHR*** ***LHCGR***	rs11692782 rs7371084 rs4953616	Case-control	Folliculogenesis Ovulation	Bahraini Arab	Differential association of LHCGR and FSHR variants with PCOS due to racial/ ethnic contribution	(30)
***FSHR***	Ala307Thr polymorphism	Cross-sectional	Folliculogenesis	Egyptian	Ala307Thr	(43)
***FSHR***	Thr307Ala and Asn680Ser polymorphism	Meta-analysis	Folliculogenesis	Different	Asn680Ser	(47)
***FST***	rs1423560 rs3797297 rs11745088 rs3203788 rs1062809 rs1127760 rs1127761	Case-control	Development of ovarian follicles Antagonist to aromatase activity Specific inhibitor of FSH	Caucasian	SNP rs3797297 associated with androgenic markers	(54)
***FST***	D5S474 D5S623 D5S822	Case-control	Follicular development	Caucasian	ND	(55)
***FST***	Presence of mutations	Case-control	Follicular development	South Indian	ND	(56)
***GDF9***	c. 1–8C>T 199A>C 205C>T 646G>A 1353C>T 398–39C>G 447C>T 546G>A 436C>T 588A>C 712A>G 1283G>C 392–393insT 1268–1269delAA 307C>T 362C>T 1121C>T 1360C>T	Case-control	Early follicle growth and fertility	Chinese Han	c.15C>G c.118T>G c.133A>G c.1025A>T c.1275C>A	(132)

* In this table, in addition the name of genes, type of evaluated polymorphism of genes, type of study, physiologic function affected by genes, studied population, and type of single nucleotide polymorphisms or polymorphisms that are associated with PCOS, have been described.

† Abbreviations: *ESR*, estrogen receptor; *FSH*, follicle stimulating hormone; *FSHR*, follicle stimulating hormone receptor; *FST*, follistatin; *GDF9*, growth/differentiation factor 9; *GnRHR*, gonadotropin releasing hormone receptor; *GWAS*, genome-wide association study; *LH*, luteinizing hormone; *LHCGR*, luteinizing hormone/chorionic gonadotropin receptor; *ND*, no data; *TSH*, thyroid stimulating hormone

**Table 2:** Candidate genes involved in etiology of polycystic ovary syndrome (PCOS) related to steroid hormones biosynthesis and functions

**Gene** *	**Genetic marker (s) **	**Type of study**	**Physiologic function**	**Studied population**	**Type of polymorphism**	**Reference**
***CYP17A1*** †	-600C>A -34C>T +723G>A +2612T>C +4259C>T +4444C>G +4994C>T	GWAS	Steroidogenesis	Korean	Overexpression of A2A2 genotype of the CT variant at -34 bp	(133)
***CYP17A1***	C/T polymorphism	Cross-sectional study	Steroidogenesis	Indian	No association with IR but associate with androgen levels	(102)
***HSD17B6*** ***GATA6***	rs898611	Case-control	Steroidogenesis A transcription factor for regulation promoter of CYP17 and CYP11A genes	Caucasian	rs898611	(134)
***HSD17B6***	rs898611	Case-control	Steroidogenesis	Alabama	rs898611	(111)
***CYP19***	rs2470152	Case-control	Steroidogenesis	Chinese	No association	(82)
***CYP11a***	5′ UTR pentanucleotide repeat polymorphism	Case-control	Steroidogenesis	Samaritan	5′ UTR pentanucleotide repeat	(91)
***CYP11a***	Microsatellite (TTTA)n polymorphism	Case-control	Steroidogenesis	South India	(TTTA)n repeat	(96)
***CYP11a***	Nine SNPs	Case-control	Steroidogenesis	Chinese Hainan	“GG” of rs4887139 and genotype “CC” of rs4886595	(89)
***CYP11a***	Pentanucleotide repeats (TTTA)n	Meta-analysis	Steroidogenesis	Different	(TTTA)n repeat polymorphism	(98)
***CYP19***	(TTTA)n polymorphism in intron 4	Case-control	Steroidogenesis	Chinese	No association	(81)
***11β-HSD1***	rs12086634	Case-control	Adrenal Steroidogenesis	Caucasian	G allele	(135)
***CYP19*** ***CYP17a***	CYP19 D10S192	Case-control	Steroidogenesis	Caucasian	ND	(55)
***CYP17a***	-34T/C polymorphism	Case-control	Steroidogenesis	Chinese Han	Association with testosterone level and HOMA-IR	(101)
***SHBG***	(TAAAA)n repeat polymorphism	Case-control	Steroidogenesis	Greek	Greater frequency of longer (TAAAA)n alleles	(79)

* In this table, in addition to the name of genes, type of evaluated polymorphism of genes, type of study, physiologic function affected by genes, studied population, and type of single nucleotide polymorphisms or polymorphism that are associated with PCOS, have been described.

† Abbreviations: *CYP*, cytochrome P450; *GATA*, transcription factors that bind to the DNA sequence (A/T) GATA (A/G); *HSD*, hydroxysteroid dehydrogenase; *IR*, insulin resistance*; ND*, no data; *SHBG*, sex hormone-binding globulin; homeostatic model assessment for insulin resistance (HOMA-IR).

***Gonadotropin secretion and actions***

Unlike the name, PCOS is probably due to impaired neuronal pathways in the brain that control the hypothalamic-pituitary-ovarian (HPO) axis (24). Ovarian functions in most mammalian are regulated by the small group of neurons localized in the preoptic area of hypothalamus, named GnRH neurons (24). The release of GnRH neuropeptide from the axon terminal of neurons into median eminence and portal vein leads to secretion of gonadotropins from the adenohypophysis gland, which in turn mediates ovarian folliculogenesis and steroidogenesis (25). FSH is responsible for stimulating the growth of follicles in the ovary, which naturally applies this effect by binding to FSH receptors on granulosa cells. If the release of FSH decreases for a long time, follicular maturation and subsequently ovulation does not occur and leads to subfertility. These immature follicles eventually form small cysts in the ovary (26, 27).

On the other hand, LH stimulates follicular growth, steroidogenesis, and formation of corpus luteum (28). Ovulation is the result of LH surge (29). The LH actions are carried out via binding to high affinity LH receptor and luteinizing hormone/chorionic gonadotropin receptor (LHCGR), which also serves as the receptor of human chorionic gonadotropin (hCG) (30). Unsuitable secretion of gonadotropins is main attribute of PCOS. Women with PCOS showed high concentrations of LH, and have high and low levels of LH and FSH, respectively; the 2/1 to 3/1 ratios usually were expressed for abnormal gonadotropin release (31).

The prominent neuroendocrine abnormalities involved in PCOS are an elevation of frequency and amplitude of GnRH release, which is reflected by LH secretion and in fact it is the main pathophysiological component of PCOS (8). Effective mechanisms for increasing pulse frequency and amplitude of LH in PCOS are not well understood, but four hypotheses have been suggested that explain the impact of peripheral hormones on brain function in pathogenesis of PCOS (Figure 3). The first hypothesis is the increase of circulating insulin level (hyperinsulinemia) that elevates the activity of GnRH neurons or pituitary responsiveness to GnRH. The second hypothesis is the low levels of serum progesterone that is followed by anovulation in PCOS conditions, which eventually removed the influence of negative feedback by progesterone on GnRH release. The third hypothesis is hyperandrogenism that changes the setting up of critical neuronal circuits for negative feedback of steroid hormones (24). The recent mentioned hypothesis seems play a serious role on the function of GnRH pulse generator that reduces the activity of GnRH inhibitors such as GnIH or its counterpart in mammals, RFRP-3. RFRP-1 and RFRP-3 neuronal cell bodies are located in the dorsomedial nucleus of the human hypothalamus and axonal projection that reach to preoptic area and median eminence (32). The mRNA expression of hypothalamic RFRP-3 neuropeptide is reduced in rats after induction of PCOS by continuous light (9). To the best of our knowledge to date, there is no research on alteration of GnIH neuronal activity in the human PCOS conditions. 

The neuroendocrine dysfunctions in PCOS are well-established; hence, the evaluation of genetic variants involved in HPO axis and their association with PCOS is valuable. In recent years, several studies in different ethnicity cohort were conducted for examination of PCOS risk genes that are related to HPO axis. Contrary to *GnRH* and its receptor (*GnRHR*) genes, FSH and LH receptor (*FSHR/LHCGR*) genes are known in the previous studies as PCOS risk susceptibility locus, regardless of racial differences (30). In addition, both *FSHβ* and *FSHR* genes are associated with PCOS risk in women, which suggest the importance of neuroendocrine pathway in PCOS pathogenesis (33).

The HPO axis is the main regulator of reproduction in females and some proteins coding genes in this axis may involve in the pathophysiology of PCOS (Table 1). They include *GnRH*, *GPR147*, *GnRHR*, *FSHβ*, *LHβ*, *FSHR*, *LHCGR*, *ER*, *PR, FST* and *RFRP/GnIH*. In following, effective genes on neuroendocrine pathogenesis and gonadotropin secretion hypothesis of PCOS are described in details.

***GnRHR gene***

The GnRH neuronal decapeptide for performing its functions binds to gonadotrope cells of anterior pituitary through GnRH receptor (GnRHR) (53). The *GnRHR* gene encodes GnRH receptor that belongs to G-proteins coupled-receptor family. After binding to its receptor, GnRH interacts with G-proteins and activates phosphatidylinositol-Ca^2+^ second messenger system, which ultimately leads to FSH and LH release (34). Due to the inconsistency of gonadotropin secretion in PCOS, evaluation of *GnRH* expression, mutation and single nucleotide polymorphism (SNP) are valuable. Molecular analysis of human genome showed unlikely that mutation in *GnRHR* gene would be the cause of PCOS formation (54). Also, the 3’-UTR rs1038426 variant in *GnRHR* gene was associated with PCOS phenotypic features, which include changes in insulin concentration during glucose tolerance test, insulin sensitivity index, and serum thyroid concentration (35). In a recently conducted GWAS on families with three sisters with PCOS diagnosis, rs104893836 variants in the first exon of the *GnRHR* gene were homozygous in three patient sisters and heterozygous in their parents (34). Furthermore, cross-talk between *GnRH* signaling and thyroid-stimulating hormone (TSH) release was documented and the role of *GnRH* signaling in glucose metabolism and insulin secretion was shown; so, genetic variations of *GnRHR* likely contribute in PCOS phenotypic expression (35). Taken together, investigations suggest that genetic alterations in *GnRH* and its receptor (*GnRHR*) genes can interfere with PCOS. Although, susceptible variants as a risk factor for PCOS in this gene have not yet been determined.

*FSHβ gene*

Commonly, investigators believe that the causes of PCOS are the elevation of LH/FSH ratio, insulin resistance and exposure to androgens during development (38, 55). The *FSHβ* gene encodes the beta subunit of FSH, which determines the specificity of this hormone (56). In a GWAS, it was found that SNP rs11031006 in the 11p14.1 chromosome in *FSHβ* region was strongly associated with PCOS and increased LH levels in Chinese women (38). There was also a higher risk for the allele A genotype in rs11031010 variant of *FSHβ* gene, which was associated with higher levels of blood LH concentration, and irrespective of ethnicity, it has been observed in European and Chinese women (33). Thus, it seems that the anomaly in FSH secretion at the transcription level may be associated with abnormal LH secretion, and origin of dysregulation in secretion of these gonadotropins is same. But, gonadotropin secretion irregularities are the primary reason for hyperandrogenism and anovulation; hence, Rotterdam criteria are questionable because, Hayes *et al*. (38) considered combination of hormonal disorders, such as hyperandrogenism, and anovulation as a cause of gonadotropin dysregulation. It seems for clarification, investigation of the causative role of hyperandrogenism in occurrence of PCOS, and evaluation of its upstream and downstream pathways drives is necessary.

*FSHR gene*

The mRNA of *FSH *receptor (*FSHR*) was expressed in granulosa cells of ovary, and FSH binds to its receptors, and then exerts its biological effects through its own signaling pathway (57). The FSHR is dedicated to G-proteins coupled receptor family. The *FSHR *gene contains 10 exons and 9 introns, and the promoter region is located on 2p21 chromosome (42). Different studies have been conducted on different variants of *FSHR* gene in relation to PCOS in various populations. For instance, Ala307Thr polymorphism in *FSHR* gene was associated with PCOS in Egyptian women (47). While in a meta-analysis study, the same Thr307Ala variant did not show any association with PCOS (44). In other studies, various variants of this gene include: rs6166, rs2268361-t, rs12994034 and rs11692782; in the Indian, European (with repetition in the participants from Greek women), other European regions and Bahraini Arab women polymorphisms at this gene were associated with PCOS (23, 30, 45, 46). In addition, Asn680Ser polymorphism in a meta-analysis study was related to PCOS (48). Overall, recent studies have shown that the genetic variation of *FSHR* gene regardless of race differences may be a risk factor for the PCOS. 

*LHCGR gene*

Increased serum LH concentration is a hallmark feature of PCOS. The *LHCGR* gene encodes receptors for both LH and hCG, and is expressed primarily in granulosa cells of preovulatory follicles in the final stages (19). SNPrs7371084 variants are negative and SNPrs4953616 is positive, which were associated with PCOS in Bahraini Arab women (30). Moreover, there was no significant association between variants of rs13405728 and lucas 2p16.3 and *LHCGR* gene (40, 41). Given that, heterogeneity of PCOS will affect the risk of PCOS development (30). Regarding the non-uniformity of the data obtained from the genomic study of *LHCGR*, it seems that racial background is effective in creating these differences. Therefore, the related continuation and independent ethnic research can be useful in verifying the relationship between the variants of gonadotropin receptors and increased risk of PCOS (30). Capalbo *et al*. (59) reported that the 312N variant in *LHCGR* gene may be a risk factor for PCOS and increased the risk of PCOS development in the Sardinian population by 2.7 fold. So, due to possible role of *LHCGR* gene in PCOS development, it is strongly recommended to evaluate their association in different populations.

*FST gene*

Follistatin (*FST* gene) is an Activin-binding glycoprotein, which is expressed in numerous tissues and can be involved in PCOS (60). The main action of follistatin is the regulation of activin activity (61), which, by binding to activin, neutralizes its effect on stimulation of FSH production and also acts as an antagonist of aromatase activity (62). Regarding follistatin actions, it can play a role in some of the major characteristics of PCOS, such as decreasing serum FSH levels, disrupting follicular development, and increasing the production of androgens (49). In a study on effective candidate genes in incidence of PCOS, genetic variation of the *FST* gene, unlike the insulin receptor (*INSR*) gene, was not associated with PCOS (50). However, although the association of SNPrs3797297 on *FST* gene was detected with androgenic indices such as SHBG concentration and free androgen index (FAI), it was not confirmed as a susceptibility gene for PCOS (49). In the study of the relationship between PCOS and *FST* gene in Indian women, no mutation was found in any of the exons of this gene (51). Therefore, the genetic evaluation of follistatin should be performed with regard to its association with androgenic characteristics of PCOS and regardless of racial differences, because the inconsistency of previous findings is mainly due to ethnic background of participants.

*RF-amide related peptide /Gonadotropin inhibitory hormone (GnIH)*

Releasing of GnRH in the brain can be directly inhibited by GnIH (63). RF-amide related peptide (RFRPs) are a peptide family and orthologue of GnIH in the mammals, and among the all members of RFRPs, RFRP-3 has been reviewed for its several reproductive functions (64). In all vertebrate species from fish (65) to human (32), the GnIH/RFRP peptides inhibit the gonadotropins secretion. Neuronal cell bodies of RFRP are present in the dorsomedial hypothalamus (DMH) (66-68). In addition, in the arcuate nucleus (ARC), paraventricular nucleus (PVN), preoptic area (POA), and periventricular nuclei (PeN) in rodents, ruminants and primates the RFRP neuronal bodies have activities (32, 67, 69-75) and develop their fibers to the other part of brain (67, 74, 75).

During estrous cycle of rodents, GnIH/RFRP neurons have regulatory role. During diestrous and estrous phases, the number of RFRP-3-ir neurons was more than early estrus and proestrus phases (66). In addition, the opposite expressions of *kiss1* and *GnIH* mRNAs in the hypothalamus of rats during different stages of estrous cycle have been shown (76). This opposite expression of *kiss1* and *GnIH* mRNAs in the hypothalamus has also been shown during malnutrition condition, pregnancy and lactation in female rats (77-79). RFRP-3/neuropeptide VF precursor (NPVF), cleaved from a preproprotein, is encoded by the *NPVF* (or *RPFP*) gene (80). In our previous study, induction of PCOS by continuous light reduced the mRNA expression of RFRP-3 neuropeptide in rats but did not have effect on kisspeptin expression (9, 81). However, it does not appear that the ovarian RFRP-3 has effect on PCOS.

**Steroid hormones biosynthesis and function**

Cytochrome P450 side chain cleavage enzyme is encoded by the *CYP11A* gene (17). Since overproduction of androgens is a major component of PCOS, identification of effective mechanisms for increasing the biosynthesis of androgens in the ovary and adrenalin in PCOS conditions is necessary to complete the understanding of steroidogenesis pathway (Figure 4).

Overproduction of androgens in PCOS patients can be mainly ovarian origin and in 20 to 30% of patients, its source is adrenal (82). The first reason for androgen overproduction in the ovary is an increase in pulses of LH secretion, which affects theca interna cells and induces androgen synthesis (8). In addition to neuroendocrine mechanism of androgen overproduction, the effect of peripheral hormones should also be taken into the account. Insulin resistance and elevation of circulatory insulin (hyperinsulinemia) that has been shown in PCOS patients leads to stimulation of androgen synthesis in adrenal and ovary via various mechanisms (11). These mechanisms include: a, increase in LH secretion, b, raise of adrenal to adrenocorticotropic hormone (ACTH), and sensitivity of ovarian theca cells to LH, c, reducing the levels of insulin-like growth factor-1 (IGF-1) binding protein (IGF-1BP) and upregulation of IGF-1 receptors in ovaries and d, inhibition of synthesis of SHBG in liver (11). 

Hyperinsulinemia directly affects ovarian theca cells through the mediation of IGF-1, or by diminished production of IGF-1BP in the liver and increases IGF-1 and IGF-2 levels. Hyperinsulinemia stimulates cytochrome P450c17α, which ultimately reinforces androgen production in ovary and adrenal, as well (83). Alteration of genetic variants in catalytic enzymes of steroidogenesis pathway as well as the SHBGs can also lead to an increase of androgen synthesis by altering their expression (84, 85). Therefore, in order to identify the risk factors of PCOS, genetic variants of genes-mediated steroidogenesis pathway have been evaluated in different studies (Table 2).

Biochemical examination of serum androgens including total testosterone, free testosterone, SHBG, androstenedione, 17-hydroxy progesterone (17-OHP) and dehydroepiandrosterone sulfate and assessing the free androgen index (FAI = (total testosterone / SHBG) × 100) is one of the ways for diagnosis of PCOS in women (1). The origins of PCOS hyperandrogenemia are the steroidogenic cells of ovary and adrenal, which in both places the effective enzymes for steroidogenesis are similar. In addition, biological impacts of androgens are mediated by androgen receptor (AR), and SHBG regulates the serum level of free androgen; so, all of these may involve in the PCOS pathophysiology. In below, these genes and their association with PCOS are described. 

*CYP19A1 gene*

This gene encodes the aromatase enzyme, which converts androgens into estrogens. Reducing aromatase activity in the ovarian follicles leads to accumulation of androgens that can be observed in PCOS. There is a positive correlation between this gene and the incidence of PCOS (7). In fact, the androgen excess in addition to hyperinsulinemia may be involved in hypertension by stimulating the expression of aromatase in adipose tissue (98). Aromatase contributes to the estrogen synthesis and androgen metabolism, thus in hypertension, the coincidence of metabolic disorder and PCOS may be observed because it is linked to hyperandrogenism and insulin resistance (98). In research by Xu *et al*. (95), polymorphism *(TTTA)n* in *CYP19* gene showed that patients with PCOS had shorter alleles in this tandem repeat. The TC genotype of heterozygous *CYP19* gene inhibits aromatase activity resulting in hyperandrogenism in PCOS patients; but, it cannot be an etiological factor in PCOS (90). In the study by Xita *et al*. (99), it was indicated that the presence of short *CYP19 (TTTA)n* alleles can be effective in this phenomenon. SNP of rs2414096 in *CYP19* gene may be susceptibility factor to PCOS in different populations (100-104). In fact, this SNP is more related to hyperandrogenism, and via reducing aromatase activity, is involved in hyperandrogenic phenotype and PCOS development (100, 103, 104). Relying on various studies representing the pivotal role of *CYP19* in hyperandrogenism, it appears that this gene by mediating androgen biosynthesis may be a susceptible gene in PCOS development.

*CYP11A gene*

*CYP11A* is the side chain cleavage (P450scc) enzyme encoding gene, the rate limiting enzyme of steroidogenesis pathway, which catalyzes the first step of steroid biosynthesis by conversion of cholesterol to pregnenolone (7). The P450scc enzyme is mostly expressed in steroidogenic tissue including ovaries, testes, adrenal cortex, and placenta (93). PCOS is mainly associated with androgen excess (93). Allelic variants of *CYP11A1* *(TTTTA)n* gene is associated with PCOS and high/low serum testosterone level (105). Also, variation in *CYP11A* gene may be involved in hyperandrogenism etiology (91). Gaasenbeek *et al*. (106) found that the linkage and association between *CYP11A* gene and PCOS are not enough strong. Repeat polymorphism *(TTTTA)n* in promoter region of *CYP11A* gene that is associated with PCOS is reported in Spanish, Chinese, Korean, and Indian women with PCOS (92-107-110). The results of meta-analysis showed an association between pentanucleotide repeat polymorphism at *CYP11A1* promoter and PCOS; however, more research is needed to confirm such association (94). So, the variations of *CYP11A* gene depend on various racial backgrounds or interfering with other genes and environment. Given the key role of this gene in rate limiting reaction of steroidogenesis, it cannot be ignored; however, there is not enough evidence that *CYP11A* gene is a risk factor for PCOS.

*CYP17A1 gene*

This gene encodes the P450cytochrome 17 (P450c17) enzyme, which has a dual role of 17α-hydroxylase and 17-lyase. This enzyme catalyzes the transformation of pregnenolone into 17-hydroxypregnenolone and of progesterone into 17-hydroxyprogesterone and also produces dehydroepiandrosterone (DHEA) (111). The activity and expression of P450c17 enzyme is increased in ovarian theca cells of women with PCOS (112). The *CYP17A1* gene may affect PCOS pathogenesis via the impact on serum testosterone levels and homeostatic model assessment for insulin resistance (HOMA-IR) (97). Although, the *CYP17A1* gene has no direct effect on PCOS susceptibility, its interaction with other susceptibility genes and contribution in polygenic conditions of PCOS (97) may be important in PCOS etiology. But, in a study by Banerjee *et al*. (87), it is suggested that the *CYP17A1* gene could not be linked to insulin resistance and it is associated with androgen excess in non-obese PCOS Indian women. In addition, increased expression of *LHCGR* and *CYP17A1* in human polycystic ovaries’ theca cells has been observed (113). Thus, confirmation of the role of *CYP17A1* in local and extragonadal abnormal steroidogenesis in PCOS needs more researches. 

*HSD3B gene*

This gene encodes 3β-hydroxysteroid dehydrogenase (3βHSD) enzyme, which catalyzes the conversion of Δ5 to Δ4 steroids (7). Type 2 3βHSD iso-enzyme is expressed in adrenal, ovary, and testis. Deficiency of this enzyme in hyperandrogenic females is related to insulin resistance of the PCOS (114). Doldi *et al*. (115) reported reduced expression of 3βHSD in granulosa cells of ≤10mm and ≥16mm follicles in polycystic ovaries compared to normal ovaries. The mild hormonal change of *HSD3B* deficiency in the hyperandrogenic female is related to metabolic phenotype of PCOS insulin resistance (116). There is little information from *HSD3B* gene polymorphism and association with PCOS, so clearing the role of its gene needs more researches. The genetic variation of this gene likely does not seem to play a role in PCOS development.

*HSD17B gene*

The protein derived from this gene called 17β-hydroxysteroid dehydrogenase (17βHSD) that catalyzes the conversion of androstenedione to testosterone (114). It was hypothesized that deficiency of this enzyme results in menstrual irregularities due to the accumulation of androstenedione (117). SNP -71G in type 5 of 17βHSD as a pragmatic polymorphism is due to androgen excess in some PCOS patients (117). In a study by Marioli *et al*. (118), it was suggested the -71G*HSDB5 *might be associated with hyperandrogenemia and biochemical hyperandrogenism, but it could not be the major effective part in the pathogenesis of PCOS (118). Also, the significant association between SNP rs1937845 of *HSD17B5* and PCOS risk in Chinese women were reported, but elucidating the exact molecular mechanism of the role of this polymorphism in PCOS hyperandrogenism needs further study (119). The intrinsic origin of ovarian steroidogenesis was increased due to the expression of *HSD17B6* in theca cells of follicles in polycystic ovaries (120). SNP rs898611 in *HSD17B6 *gene was associated with phenotypic metabolic features of PCOS (89). So, *HSD17β* enzyme is involved in PCOS hyperandrogenism, but how much contribute to the onset of hyperandrogenism cannot be precisely determined.

*AR gene*

This gene encodes an androgen receptor that is located on Xq11-12 chromosome. Naturally, many of the effects of hyperandrogenism in patients with PCOS are mediated through this receptor. The relationship between serum testosterone elevation and presence of longer CAG sequences in the *AR* gene remain uncertain (121). Overexpression of *AR* was observed in the granulosa cells, luminal, and glandular epithelium of endometrium in polycystic ovaries compared to normal ovaries (122, 123). Hyperactive ARs at the level of GnRH pulse generator, granulosa cells, skeletal muscles, or adipocytes sense testosterone and dihydrotestosterone as a biochemical hyperandrogenic status, and by binding to theses androgens act their functions in these places; thus, it seems that there is relationship between the site of *AR* and PCOS pathogenesis (124). Shorter CAG repeat polymorphism in exon 1 *AR* gene was associated with an increase of its activity and PCOS pathogenesis (124, 125). The association of shorter CAG repeats of *AR *with PCOS was demonstrated in *in vivo* and *in vitro* researches. This polymorphism leads to the upregulation of AR and increment androgen sensitivity in PCOS patients (126). Another suggested mechanism regarding this association is an increase in the intrinsic androgen production (127). However, some researcher believed that androgen repeat CAG cannot be the main genetic determinant in PCOS phenotypes (128), but some others expressed that CAG repeat polymorphism could be the modulator genetic marker in PCOS heterogeneous features and even is related to metabolic consequences (129). So, demonstration of *AR* gene as a risk factor for PCOS needs further investigations. 

*SHBG gene*

The protein derived from this gene that is known as the SHBG actually regulates the access of tissues to androgens. Reduction of *SHBG* levels is an attribute of hyperandrogenic women that increases the release of free androgen to tissues (130). Serum levels of *SHBG* in PCOS women were higher than normal women, so the circulating free androgens are increases, and tissues are more exposed to androgens (85). The lower level of *SHBG* in PCOS patients leads to the expression of *SHBG* coding gene as a candidate in PCOS etiology. Also, the lower concentration of *SHBG* in the placenta during fetal development has contributed to PCOS fetal programming (13). Positive correlation of hyperandrogenism with *(TAAAA)n* polymorphism in *SHBG* gene promoter has been confirmed (130). The *(TAAAA)n* pentanucleotide repeat influences the levels of *SHBG*, and shorter length of repeat is related to more transcription of gene (131). Variants of *SHBG* gene were also associated with metabolic syndrome in obese women and also Mediterranean women with PCOS, suggesting that the *SHBG* gene may be a risk factor for PCOS (132, 133). Given the important role of *SHBG* in the incidence of PCOS-induced hyperandrogenism, it is likely to be a susceptibility gene in PCOS.

*StAR gene*

A steroidogenic acute regulatory protein called StAR is a type of transporter protein that transmits cholesterol into the mitochondria of steroidogenic cells. In PCOS patients, the androgen synthesis increases and StAR also carries out the first step of steroidogenesis in the ovary and adrenal (134). So, genetic variation of *StAR* gene may be involved in PCOS hyperandrogenism etiology. In a study by Nazouri *et al*. (135), no relation between seven SNPs of *StAR* genes was observed in Iranian PCOS women. Considering the increased intra-ovarian androgens hypersecretion, StAR-ir increased in theca cells of cystic follicles (134). However, the cholesterol availability contributes to increased androgen synthesis in the PCOS condition (134). The further research is necessary to identify the effect of *StAR* in hyperandrogenism of PCOS. 

**Conclusions and Future outlooks**

Taken together, the hyperandrogenism is the potent concept about PCOS etiology. The previous pathways before the hypersecretion of androgens such as hypothalamic-pituitary-gonadal (HPG) axis disturbances are valuable for investigation. Pathophysiological functions of genes that are primarily responsible for the synthesis of proteins have role in PCOS before hyperandrogenism. The final functions of these genes include gonadotropin secretion and actions as well as steroid hormones biosynthesis and functions. Between the reported genes in this article, the genes that have shown their effects on gonadotropin secretion and actions in PCOS women are *GnRHR*, *FSHβ*, *FSHR*, and *LHCGR*. Also, between the reported genes, the genes that have effects on steroid hormones biosynthesis and functions in PCOS women include* CYP19A1*, *HSD17B*, *AR *and *SHBG*.

Each of the two hypotheses mentioned above can be the initiator of PCOS, but evaluations of these hypotheses are rather difficult, because they may be affected by other initial factors. As for hyperandrogenism, by applying fetal, neonatal, and prepubertal androgenic treatments on animal models, it can be possible to induce PCOS features. Another physiological pathway as the primary cause of the syndrome can be examined by disrupting the HPO axis using animal models, animal genetic manipulation models or transgenic models. Another way is finding genes with the highest dependency on PCOS and then finding co-expressing genes with them, or their underlying physiological pathways. So, study on PCOS has opened the way for many researches in future.
